# The social functioning in dementia scale (SF-DEM): Exploratory factor analysis and psychometric properties in mild, moderate, and severe dementia

**DOI:** 10.1016/j.dadm.2018.11.001

**Published:** 2019-01-02

**Authors:** Jessica Budgett, Anna Brown, Stephanie Daley, Thomas E. Page, Sube Banerjee, Gill Livingston, Andrew Sommerlad

**Affiliations:** a Division of Psychiatry, University College London, London, UK; b Camden and Islington NHS Foundation Trust, St Pancras Hospital, London, UK; c School of Psychology, University of Kent, Canterbury, UK; d Centre for Dementia Studies, Brighton and Sussex Medical School, University of Sussex, Brighton, East Sussex, UK

**Keywords:** Psychometrics, Factor analysis, Social behaviour, Dementia, Behavior Rating Scale

## Abstract

**Introduction:**

The psychometric properties of the social functioning in dementia scale over different dementia severities are unknown.

**Methods:**

We interviewed 299 family carers of people with mild, moderate, or severe dementia from two UK research sites; examined acceptability (completion rates); conducted exploratory factor analysis; and tested each factor's internal consistency and construct validity.

**Results:**

Of 299, 285 (95.3%) carers completed questionnaires. Factor analysis indicated three distinct factors with acceptable internal consistency: *spending time with other people*, correlating with overall social function (r = 0.56, *P* < .001) and activities of daily living (r = −0.48, *P* < .001); *communicating with other people* correlating with activities of daily living (r = −0.66, *P* < .001); and *sensitivity to other people* correlating with quality of life (r = 0.35, *P* < .001) and inversely with neuropsychiatric symptoms (r = −0.45, *P* < .001). The three factors' correlations with other domains were similar across all dementia severities.

**Discussion:**

The social functioning in dementia scale carer version measures three social functioning domains and has satisfactory psychometric properties in all severities of dementia.

## 1 Introduction

Decline in social functioning, defined as “how individuals associate and interact, both in society at large and their own personal environment” [1], accompanying cognitive deterioration is part of the diagnostic criteria for dementia [2]. Such changes can occur in the early stages of a number of conditions causing dementia, including Alzheimer's disease [3] and frontotemporal dementia [4]. Putative mechanisms include disruption to the amygdala and frontal cortex network [5] or deficits in emotion recognition [6] or theory of mind [7]. Changes in social function, such as reduced engagement in close relationships or lack of interest in previously valued hobbies, are distressing to people with dementia [8], [9] and can be upsetting and stressful for family carers [10] especially when the person with dementia lacks awareness of these changes [11]. However, social change is not routinely asked about or reported as a symptom in clinical settings [12].

The social functioning in dementia scale (SF-DEM) is a self- or carer-reported scale measuring the social functioning of a person with dementia, completed following a face-to-face structured interview. It has been found to have high acceptability and reliability and moderate concurrent validity against a single item rating overall social functioning [13] in initial field testing; this study included 30 dyads of people with mild dementia and their family carers from a single research site. Concurrent validity and test-retest reliability for assessing social function in mild dementia were better for carer-rating than self-rating. However, its psychometric properties in people with more severe dementia have not been established and its factor structure has not been explored. Factor analysis allows identification of latent constructs within a set of measured variables; derived factors can be scored separately for future studies, and their reliability and validity should be established [14]. We therefore conducted a multicenter study examining SF-DEM's acceptability, reliability, and validity for testing the social function of people with mild, moderate, and severe dementia, as rated by their family carers and explored SF-DEM's factor structure to identify the underlying relationship between its measured domains.

### 1.1 Aims

We aimed to

1. test SF-DEM's acceptability for assessing social function in people with dementia of any severity
2. examine the underlying factor structure of SF-DEM using EFA
3. test the internal consistency and construct validity of the scale and its derived factors against measures of a person with dementia's quality of life, symptoms, and dementia severity. We hypothesized that social functioning would be correlated positively with better quality of life and that social functioning would be inversely correlated with dementia severity and neuropsychiatric symptoms.

## 2 Methods

This cross-sectional study of family carers of people with dementia was nested in the CDEMQOL study (Alzheimer's Society project grant: 234 AS-PG-14-017), and the South Central–Hampshire A Research Ethics Committee gave ethical approval (15/SC/0605).

### 2.1 Setting

We recruited study participants from clinical services based in three UK National Health Service mental health trusts in Sussex and North London, which provide dementia assessment and management services; Join Dementia Research network [15], a National Institute for Health Research resource for people with dementia, carers, or healthy volunteers to register their interest in participating in research studies; registers of carers who had previously consented to be contacted about other studies; voluntary sector carers groups; and self-referrals.

### 2.2 Participants

We recruited family carers, aged over 18 years, of people with dementia of any subtype and severity (diagnosed in clinical services), who defined themselves as having a caring role for the person with dementia, and having primary responsibility for the well-being and decisions for the person with dementia, although others may have been involved in the process. We excluded paid carers or those who were unable to speak English sufficiently to complete the assessment.

### 2.3 Procedures

Trained researchers conducted a single semistructured interview with family carers at their homes or in a university or hospital site according to their preference, between June 2017 and January 2018.

### 2.4 Measures

SF-DEM [13] is a 20-item interviewer-administered questionnaire, which has patient- and carer-rated versions. In this study, we used the carer-rated scale. Seventeen items about different aspects of social function are scored using a Likert scale (0 to 4 indicating frequency of each social function domain; “never” to “very often”) with a higher score indicating better social function. Three unscored summary questions assess overall impression of social function, recent change, and willingness to make future social changes.

We recorded the age, sex, ethnicity, marital status and level of education of the person with dementia and carer and their relationship, the living situation of the person with dementia, and the dementia subtype. We also completed the following validated carer-rated measures of the person with dementia's symptoms and dementia severity.

1. Bristol Activities of Daily Living Scale (B-ADL) [16]—measures ability in 20 basic and complex activities of daily living (ADLs); higher score indicates worse ADL function.
2. Neuropsychiatric Inventory (NPI) [17]—records the presence of 12 neuropsychiatric symptoms of dementia; higher score indicates more severe symptoms.
3. DEMQOL-Proxy [18]—31-item dementia-specific health-related quality of life interviewer-administered questionnaire answered by the carer; a higher score indicates better quality of life.
4. Clinical Dementia Rating Scale (CDR) [19]—characterizes level of cognitive and functional impairment to define dementia severity (from 0.5 = very mild to 3 = severe).

### 2.5 Analysis

We completed all statistical analysis using SPSS (version 23). Ten percent of the collected data were checked through double entry for any disparities. We first described the demographic and clinical characteristics of the study sample.

We assessed SF-DEM's acceptability and feasibility in mild, moderate, and severe dementia. As missing data suggest that participants were unable or unwilling to answer questions, we reported the frequency of missing data according to level of dementia severity; we decided *a priori* that individual items would be classed as acceptable if ≥90% completion. We also recorded the time taken to complete the scale.

Although SF-DEM was developed to measure the interaction of people with dementia with society in general and their own personal environments, we did not have specific hypotheses about the factor structure of the developed measure. We therefore used EFA to empirically establish the common sources of variance underlying the item responses [14], on the 17 scored items of the SF-DEM scale to identify the latent factor structure of SF-DEM and determine if SF-DEM scores reflect a single “social functioning” domain. We used unweighted least square extraction method, which is an acceptable method if there are fewer than five response options [20]. We rotated the factors using promax (oblique) rotation [21]. The number of factors was decided by examining the scree plot and including the number of data points above the “elbow” as well as examining the model residuals [21]. We considered 0.3 as a minimum factor loading value to identify salient loadings [22]. We then evaluated the internal consistency of the identified factors by calculating the Cronbach's α coefficients and calculated the Pearson correlation coefficients between factors. The research team, which included academics from psychiatry, statistics, occupational therapy, psychology, and psychometrics, discussed whether to remove any questions, taking into account factor loading, internal consistency, and face validity.

We then created sum scores for each of the SF-DEM factors derived from the EFA and then evaluated the construct validity of each, using other carer-reported measures of a person with dementia's quality of life, symptoms, function, and dementia severity. We used Pearson to assess correlation of SF-DEM factor score totals with the summary question overall impression of current social functioning, B-ADL total score, NPI total, DEMQOL-Proxy total score, and CDR.

As secondary analyses, we repeated the validity tests stratified for the level of dementia severity as assessed by CDR. For these analyses, we examined the correlation between SF-DEM factors and the scale against which it had highest correlation.

## 3 Results

We recruited 300 study participants; one carer withdrew before completing SF-DEM, so we included data from 299 carers. Table 1 summarizes the demographic and clinical characteristics of participants and their relatives with dementia. The mean age of family carers was 63 years (standard deviation [SD] 14), and three-quarters were female. The mean age of people with dementia was 81 years (SD 8), and 60% were female. Nearly half the participants were daughters or sons of the person with dementia, and most of the others were spouses or long-term partners of the person with dementia. Alzheimer's disease was the commonest dementia subtype, and there was a range of dementia severity; over two-thirds had mild or moderate dementia.

**Table 1:** Clinical and demographic characteristics of participants

Characteristic	Carers (n = 299)	People with dementia (n = 299)
	Mean (SD), range	
Age (years)	63 (14), 21-90	81 (8), 55-98
	n (%)	
Sex		
Female	218 (73)	179 (60)
Ethnicity		
White British	253 (84.6)	241 (80.6)
White – Other	26 (8.7)	38 (12.7)
Black and minority ethnic	19 (6.7)	20 (6.6)
Marital status		
Married/cohabiting	238 (79.6)	164 (55.2)
Widowed	5 (1.7)	108 (36.4)
Single	36 (12)	4 (1.3)
Divorced/separated	20 (6.7)	21 (7.1)
Level of education		
No qualification	23 (7.7)	77 (26.6)
School (O levels/A levels)	79 (26.5)	91 (31.5)
Degree/postgraduate	145 (48.7)	75 (25.9)
Other	30 (12.8)	46 (15.9)
Living situation of carer		
Resident carer	150 (50.2)	
Nonresident carer	148 (49.5)	
Living situation of PWD		
Lives alone		53 (17.7)
Lives with others		248 (82.3)
Carer relationship to PWD		
Spouse/long-term partner	128 (42.8)	
Son/daughter	148 (49.5)	
Other	13 (7.7)	
Employment status		
Paid employment	109 (36.5)	
Unemployed/retired/full-time carer	190 (63.5)	
Dementia subtype		
Alzheimer's disease		159 (53)
Vascular		36 (12)
Other		79 (263)
Not known		25 (8.3)
Mean B-ADL score (SD)		27.85 (16.32)
Mean NPI score (SD)		24.12 (20.01)
Mean DEMQOL-proxy score (SD)		94.55 (14.29)
CDR, very mild, n (%)		31 (10.4)
CDR, mild, n (%)		108 (36.1)
CDR, moderate, n (%)		99 (33.1)
CDR, severe, n (%)		61 (20.4)

Abbreviations: SF-DEM, Social Functioning in Dementia scale; SD, standard deviation; PWD, person with dementia; B-ADL, Bristol Activities of Daily Living Scale; NPI, Neuropsychiatric Inventory; DEMQOL, Dementia Quality of Life scale; CDR, Clinical Dementia Rating scale score.

### 3.1 Acceptability and feasibility

We report the SF-DEM questions, participants' responses, and presence of missing data fully in Supplementary Appendix A. Of 299, 285 (95.3%) carers answered all 17 questions; the questions with most frequent missing responses were questions 12, 13, and 17, which were unanswered by 1% to 2% of participants. Missing data were more frequent for carers of people with severe dementia: 15 of the 23 total missing questions were from carers of people with severe dementia, but 46 of 61 (75.4%) carers of people with severe dementia completed the whole questionnaire. The full range of scores was used by participating carers for all 17 questions. The mean time taken to complete the SF-DEM was 5 minutes, and the longest completion time was 14 minutes.

### 3.2 Underlying factor structure measured by the SF-DEM

In the EFA, the first three factors explained 13.9%, 23.2%, and 11.3% of the variance, respectively. The three-factor solution, which explained 48.4% of the variance, was preferred because of the “leveling off” of eigenvalues on the scree plot after three factors (Supplementary Appendix C) and the difficulty of interpreting the fourth and subsequent factors.

The three-factor solution was rotated obliquely to yield standardized factor loadings presented in Table 2. Factor 1 comprised 7 items (domains 1-2, 4-8) with factor loadings from 0.21 to 0.70; we summarized this factor with the label “Spending time with other people.” Although item 1 had low factor loading, we decided to retain this item (“how often … has your relative seen friends or family in their own home?”) for face validity. Factor 2 comprised 6 items (domains 3, 9-13) with factor loadings from 0.48 to 0.77, and we describe it as “Communicating with other people.” Factor 3 comprised 4 items (domains 14-17) with factor loadings from 0.45 to 0.78, and we labeled this “Sensitivity to other people” because the domains reflect a lack of awareness of the needs and emotions of others. Factors 1 and 2 were weakly correlated (r = 0.38), but there was no correlation of either of these factors with factor 3 indicating that there was no overarching general factor measured by SF-DEM.

**Table 2:** Summary of exploratory factor analysis for SF-DEM: Standardized factor loadings and factor correlations (N = 285)

SF-DEM item	Factor loadings		
	Spending time with other people	Communicating with other people	Sensitivity to other people
1. Seen friends or family in own home	0.21	−0.09	−0.10
2. Gone to visit friends or family in their home	**0.58**	0.19	0.00
3. Contacted friends or family by phone or computer	0.25	**0.48**	0.00
4. Attended community or religious meetings	**0.51**	−0.10	−0.02
5. Gone shopping with friends or family	**0.49**	0.14	−0.02
6. Gone on trips or to events like cinema or talks	**0.60**	0.07	0.00
7. Gone to a café, restaurant, pub, or social club	**0.56**	−0.01	0.03
8. Exercised, walked, or played sport with others	**0.70**	−0.13	0.02
9. Started or taken part in a conversation	0.03	**0.70**	−0.13
10. Talked to you or other about their feelings or concerns	0.01	**0.54**	−0.16
11. Asked you or others about your/their feelings or concerns	0.07	**0.49**	0.10
12. Been more limited in their topics of conversation	−0.09	**0.72**	0.06
13. Found it difficult to follow conversations	−0.16	**0.77**	0.15
14. Been very outspoken about what they really think	0.09	−0.18	**0.66**
15. Been irritated at things other people have done or said	0.05	−0.04	**0.78**
16. Had an argument or shouted at other people	−0.02	0.17	**0.69**
17. Found reasons not to do things they would usually do	−0.17	0.10	**0.45**
Factor correlations (r)	Factor 1	Factor 2	Factor 3
Factor 2	0.38		
Factor 3	0.001	−0.19	

NOTE. 1) Using unweighted least square extraction method; 2) Factor loadings over 0.3 appear in bold.

### 3.3 Internal consistency

Internal consistency for each of the factors was acceptable: Cronbach's α = 0.73 for *spending time with other people*; α = 0.79 for *communicating with other people*; and α = 0.72 for *sensitivity to other people*. One item (“Seeing friends and family in their own home”) had lower item-total correlation and poorer factor loadings but removing it would reduce face validity with little improvement in the Cronbach's α score, so we chose for all items to remain in the scale.

We then calculated sum scores for the three scales and used the sum scores for subsequent analyses (scale 1 = *spending time with other people*; scale 2 = *communicating with other people*; scale 3 = *sensitivity to other people*).

### 3.4 External validity and sensitivity

Table 3 reports the correlations between the SF-DEM scales and other patient-centered domains. There was a moderate positive correlation between *spending time with other people* and overall carer-rating of social functioning (r = 0.56, *P* < .001), and *communicating with other people* and *sensitivity to other people* was weakly positively correlated with overall rating of function (Table 3). Higher scores on *spending time with other people* and *communicating with other people* were moderately associated with more independence in ADLs (r = −0.48, *P* < .001, and r = −0.66, *P* < .001, respectively). Higher *communicating with other people* and *sensitivity to other people* ratings were moderately associated with less neuropsychiatric symptoms (r = −0.36, *P* < .001, and r = −0.45, *P* < .001, respectively), and higher *sensitivity to other people* was associated with better quality of life (r = 0.35, *P* < .001).

**Table 3:** Correlation between social functioning dementia (SF-DEM) scale factor scores and patient and carer domains

Patient and carer domains	Factor 1 (spending time with others)	Factor 2 (communicating with others)	Factor 3 (sensitivity to others)
SF-DEM overall function rating	0.56 (*P* < .001)	0.29 (*P* < .001)	0.15 (*P* = .009)
SF-DEM Factor 1		0.32 (*P* < .001)	−0.03 (*P* = .61)
SF-DEM Factor 2	0.32 (*P* < .001)		−0.15 (*P* = .01)
SF-DEM Factor 3	−0.03 (*P* = .61)	−0.15 (*P* = .01)	
B-ADL total score	−0.48 (*P* < .001)	−0.66 (*P* < .001)	0.11 (*P* = .07)
NPI total score	−0.23 (*P* < .001)	−0.36 (*P* < .001)	−0.45 (*P* < .001)
DEMQOL-proxy total score	−0.04 (*P* = .51)	−0.13 (*P* = .03)	0.35 (*P* < .001)

Abbreviations: B-ADL, Bristol Activity of Daily Living Scale; CDR, Clinical Dementia Rating scale score; HADS, Hospital Anxiety and Depression Scale score; NPI, Neuropsychiatric Inventory score.

NOTE. Pearson's correlation coefficients.

**Fig. 1:**
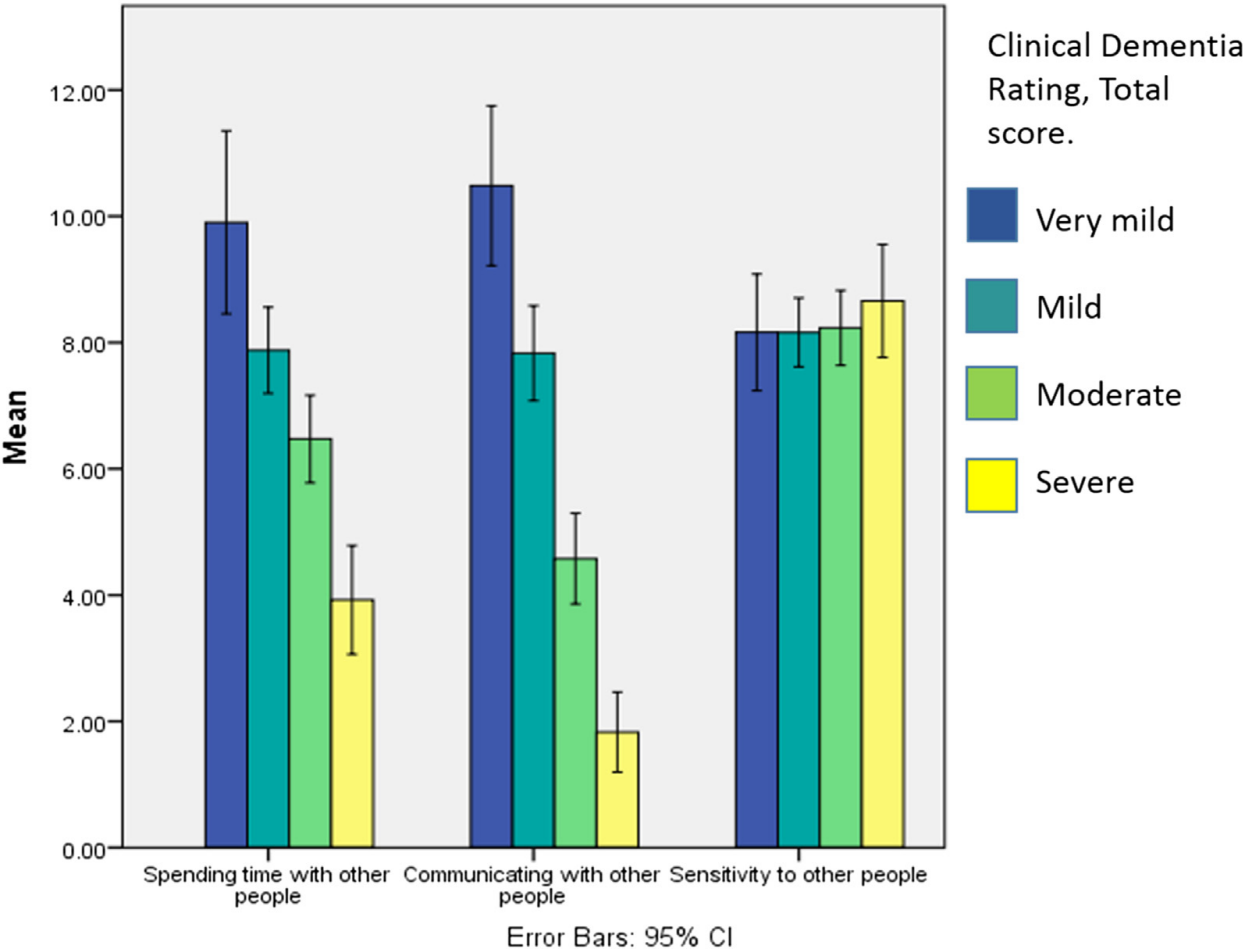
SF-DEM factor scores stratified by CDR severity. Abbreviation: CDR, Clinical Dementia Rating scale score.

Fig. 1 shows the comparison of mean SF-DEM scale scores between the levels of dementia severity measured by the CDR. The mean score for *spending time with other people* and *communicating with other people* decreased with dementia progression, and the scores were significantly different between the four levels of CDR severity as determined by a one-way ANOVA [*F* (3, 292) = 26.1, *P* < .001; *F* (3, 287) = 56.1, *P* < .001]. There was no significant difference between *sensitivity to other people* mean scores according to dementia severity [*F* (3, 289) = 0.58, *P* = .63].

In validity analyses with the sample stratified for the level of dementia severity (Supplementary Appendix B), the correlations between the three SF-DEM factors and the overall summary question of social functioning were similar across all levels of dementia severity, although correlation between *communicating with other people* score and overall social functioning rating was lower for moderate and severe dementia; correlation between *sensitivity to other people* score and overall social function rating was also lower in people with severe dementia. Correlation between *spending time with other people* score and ADL rating (B-ADL score) remained consistent for severe dementia, but coefficients were smaller for very mild to moderate categories (Supplementary Appendix B). The correlation between *communicating with other people* score and ADLs was similar across mild to severe dementia severities but lower for very mild dementia. Coefficients for the correlation between *sensitivity to other people* score and the NPI score were similar across mild to severe levels of dementia but lower in very mild dementia.

## 4 Discussion

We report results from this large multicenter study testing the psychometric properties of the carer-rated SF-DEM. These data suggest that the scale is an acceptable, reliable, and valid tool for measuring the social functioning of a person with dementia of any severity. Our EFA indicates that SF-DEM measures three distinct factors related to social functioning: *spending time with other people*, *communicating with other people,* and *sensitivity to other people* and that the corresponding sum scores had acceptable internal consistency as estimated by coefficient α. We found, as expected, that spending time with other people and communicating with other people decline with dementia progression, but this was not the case for sensitivity to other people, which varied little at differing dementia severities, possibly suggesting that this factor reflects premorbid personality [23].

We chose to test the carer-reported tool as it had higher reliability than the patient-reported tool in previous psychometric testing [13] and because we judged that people with more severe forms of dementia would be less able to accurately report on their social function due to impaired memory. Our results indicate that the carer-rated SF-DEM is an acceptable and feasible measure to complete as there were few missing data and it took only 5 minutes to complete. The 17 domains included in the SF-DEM were grouped into three scales following the EFA, and because these scales were not strongly correlated, subsequent studies should treat them as separate dimensions.

The Mild Behavioral Impairment checklist [24] assesses social behavior change in people without dementia, aiming to identify prodromal behavioral symptoms. SF-DEM's *sensitivity to others* factor assesses similar social behavioral change in people with dementia, aiming to rate severity of social function impairment, rather than as a diagnostic checklist. There are no other validated measures of social function in dementia so we are not able to assess validity by comparing the SF-DEM with another psychometrically acceptable measure. However, higher scores in all three SF-DEM scales were associated with a better rating on a question rating overall social functioning, and we used measures of ADLs function, neuropsychiatric symptoms, and quality of life with which we expected SF-DEM to be correlated. As expected, more time spent with other people and better communication with others were correlated with levels of dependence as measured by the B-ADL scale. Better communication with others was consistently associated with better ADL scores across all dementia severity, while a weaker correlation was found for spending more time with others and ADL performance. This result suggests that a person with dementia's ability to communicate is associated with greater independence in ADLs and maintaining more independence. This is likely to be because these are also markers of dementia severity and this is supported by our finding that these factor scores were lower in those with more severe dementia. Sensitivity to others, however, did not differ between dementia severity levels.

As dementia progresses, people may be less motivated to engage in social activities, have increasing problems in organizing activities, or be worried about being able to understand complex situations or getting lost. Social norms mean that tolerance of people with cognitive challenges can be low and may worsen social isolation in people with dementia [25]. People with severe dementia may display more challenging behavior and other health complications, which can result in fewer visits from friends or family members who can be distressed by these symptoms, and are more likely to move into care homes for support, which can further distance them from their support network [25]. A decline in communication as the disease progresses is seen across dementia subtypes [26]. Agitation is more common in moderate and severe dementia and can be related to physical or psychological distress [27] and a lack of social engagement [25]. Sensitivity to others, on the other hand, can vary between individuals with dementia and between different dementia types. Reduced sensitivity to others can be seen early in frontotemporal dementia and is likely to progress with disease severity [28], but frontotemporal dementia is relatively rare in our sample.

Higher sensitivity to other people was associated with less severe neuropsychiatric symptoms, such as agitation, depression, and psychosis, which may reflect that these symptoms can manifest, or be interpreted by a carer, as insensitivity in personal relationships or that not understanding others' interactions might lead to such symptoms. One longitudinal study has demonstrated that good mental health and social relationships are the key predictors of future well-being in dementia [29].

### 4.1 Strengths and limitations

This large multicenter research study is the first to evaluate SF-DEM's factor structure and test the validity of the derived factors in a large and diverse research sample. There are limitations to this study. First, although our sample included participants from two demographically differing sites, we only recruited people who were in contact with clinical dementia services or who had registered their interest in participating in research so our results may not be applicable to other populations; however, our sample included people with a range of clinical and demographic characteristics. We were also only able to include carers who could speak English so have not tested the use of SF-DEM in other languages or other cultural settings. As this study was cross-sectional, we were unable to test SF-DEM's test-retest reliability, but reliability was found to be very high previously [13]. We were also unable to test responsiveness to change, but considering that two SF-DEM factors were associated with dementia severity, it is likely that these domains would change over time. We did not test the patient-reported SF-DEM version, but there was correlation within dyads in a previous study, so expect that results would be similar for the patient-reported version in people with milder dementia. This may not be the case in more severe disease as patients develop more cognitive impairment and may have reduced insight. We did not conduct structured interviews with respondents about the acceptability and feasibility of completing the SF-DEM measure, but previous research found it to have high acceptability [13].

### 4.2 Clinical implications and future research

Social function is an important domain to consider in the diagnosis of, and interventions for, dementia, and we report for the first time that the carer-rated SF-DEM scale has acceptable validity in assessing social function of people with any dementia severity. This validated measure informs future research such as enabling intervention evaluation studies to measure social function as an important patient-reported outcome; and SF-DEM may also be a useful scale for clinical use in tracking social decline in the context of disease progression. The results of our factor analysis can guide future SF-DEM use as it indicates that three subdomains of social functioning should be used and scored separately. The correlation between two SF-DEM domains and dementia severity suggests that these domains decline with increasing severity, but future longitudinal research should test the responsiveness of SF-DEM in measuring change and examine correlates of social function change over time. In particular, future research should examine sensitivity to others over time as this study is, to our knowledge, the first to find a preserved domain throughout the range of dementia severity. Finally, considering the difference in scores between even very mild and mild dementia, such testing should include preclinical populations such as those with mild cognitive or behavioral impairment and consider correlation with the Mild Behavioral Impairment checklist [24] to examine whether changes in social function are early behavioral markers of those who will progress to dementia.

**Research in Context**

1. Systematic review: The authors reviewed the literature using traditional (e.g., PubMed) sources. The social functioning in dementia scale (SF-DEM) is the only scale specifically designed to measure social functioning in dementia. One previous study examined SF-DEM's psychometric properties in assessing social function in mild dementia, and no analysis has examined its factor structure.
2. Interpretation: SF-DEM was acceptable, valid, and reliable in people with dementia of any severity. We found in exploratory factor analysis (EFA) that it measures three distinct factors related to social functioning. The score for s*pending time with other people* and c*ommunicating with other people* was associated with dementia severity but *sensitivity to other people* was not.
3. Future directions: Future longitudinal research should test SF-DEM's responsiveness and correlates of social function change over time. Such testing should examine whether sensitivity to others changes with advancing disease and consider whether SF-DEM changes are early markers of disease in preclinical populations.

## Supplementary data

Supplementary Appendices A–C
