# Supplementary material for: The social functioning in dementia scale (SF-DEM): Exploratory factor analysis and psychometric properties in mild, moderate, and severe dementia
Source: Alzheimers Dement (Amst). 2019 Jan 2;11:45–52. doi: 10.1016/j.dadm.2018.11.001 (PMC6317324; doi:10.1016/j.dadm.2018.11.001)

Supporting information

**Appendix A: Summary of participants’ responses and scores on social functioning in dementia scale (SF-DEM)**

|  | Caregiver rated (n=299) | | | | | | |  | |
| --- | --- | --- | --- | --- | --- | --- | --- | --- | --- |
|  | Frequency (%) | | | | | | |  | |
| SF-DEM domain | **Very Often** | **Often** | | **Occasionally** | | **Never** | | | **Missing** |
| 1. | 71 (23.7) | 156 (52.2) | | 52 (17.4) | | 20 (6.7) | | | 0 |
| 2. | 6 (2) | 61 (20.4) | | 75 (25.1) | | 157 (52.5) | | | 0 |
| 3. | 21 (7) | 56 (18.7) | | 31 (10.4) | | 191 (63.9) | | | 0 |
| 4. | 14 (4.7) | 63 (21.1) | | 38 (12.7) | | 183 (61.2) | | | 1 (0.3) |
| 5. | 9 (3) | 92 (30.8) | | 58 (19.4) | | 140 (46.8) | | | 0 |
| 6. | 4 (1.3) | 33 (11) | | 53 (17.7) | | 209 (69.9) | | | 0 |
| 7. | 18 (6) | 113 (37.8) | | 76 (25.4) | | 92 (30.8) | | | 0 |
| 8. | 24 (8) | 71 (23.7) | | 58 (19.4) | | 143 (47.8) | | | 0 |
| 9. | 123 (41.1) | 57 (19.1) | | 50 (22.7) | | 68 (22.7) | | | 0 |
| 10. | 34 (11.4) | 59 (19.7) | | 70 (23.4) | | 136 (45.5) | | | 0 |
| 11. | 9 (3) | 25 (8.4) | | 61 (20.4) | | 204 (68.2) | | | 0 |
| 12. | 139 (46.5) | 63 (21.1) | | 38 (12.7) | | 55 (18.4) | | | 4 (1.3) |
| 13. | 144 (48.2) | 70 (23.4) | | 51 (17.1) | | 30 (10) | | | 4 (1.3) |
| 14. | 30 (10) | 46 (15.4) | | 62 (20.7) | | 160 (53.5) | | | 1 (0.3) |
| 15. | 30 (10) | 62 (20.7) | | 109 (36.5) | | 96 (32.1) | | | 2 (0.7) |
| 16. | 21 (7) | 32 (10.7) | | 89 (29.8) | | 155 (51.8) | | | 2 (0.7) |
| 17. | 26 (8.7) | 71 (23.7) | | 86 (28.8) | | 111 (37.1) | | | 5 (1.7) |
| SF-DEM Overall Function | **Excellent** | | **Good** | | **Fair** | | **Poor** | | |
| 1. | 7 (2.3) | | 80 (26.8) | | 93 (31.1) | | 119 (39.8) | | |
|  | **A lot better** | **A bit better** | | **No Change** | | **A bit worse** | | | **A lot worse** |
| 2. | 14 (4.7) | 26 (8.7) | | 100 (33.2) | | 94 (31.4) | | | 65 (21.7) |
|  | **Rather they do more** | | **No change needed** | | **Rather they do less** | | **Missing** | | |
| 3. | 174 (58.2) | | 116 (38.8) | | 6 (2) | | 3 (1) | | |

**Appendix B: Correlation between social functioning in dementia scale (SF-DEM) factor scores and other patient and carer domains, stratified by CDR category**

| CDR category | Measure | Factor 1 (Spending time with others) | Factor 2 (Communicating with others) | Factor 3 (Sensitivity to others) |
| --- | --- | --- | --- | --- |
| Very mild | SF-DEM Overall function rating. | -0.61 (p<.001) | -0.24 (p=.19) | -0.22 (p=.23) |
|  | B-ADL total score | -0.15 (p=.41) | -0.35 (p=.51) | 0.04 (p=.82) |
|  | NPI total score | -0.15 (p=.41) | -0.42 (p=.02) | -0.28 (p=.13) |
|  | DEMQOL proxy total score | -0.003 (p=.98) | 0.14 (p=.47) | 0.20 (p=.28) |
| Mild | SF-DEM Overall function rating Q1. | -0.53 (p<.001) | -0.24 (p=.015) | -0.24 (p=.014) |
|  | B-ADL total score | -0.17 (p=.082) | -0.38 (p<.001) | 0.06 (p=.53) |
|  | NPI total score | -0.20 (p=.034) | -0.25 (p=.009) | -0.42 (p<.001) |
|  | DEMQOL proxy total score | -0.08 (p=.44) | 0.04 (p=.66) | 0.27 (p=.005) |
| Moderate | SF-DEM Overall function rating Q1. | -0.43 (p<.001) | -0.08 (p=.43) | -0.20 (p=.05) |
|  | B-ADL total score | -0.12 (p=.23) | -0.27 (p=.007) | 0.11 (p=.89) |
|  | NPI total score | -0.03 (p=.74) | -0.26 (p=.012) | -0.61 (p<.001) |
|  | DEMQOL proxy total score | 0.01 (p=.92) | -0.26 (p=.012) | 0.44 (p<.001) |
| Severe | SF-DEM Overall function rating Q1. | -0.50 (p<.001) | -0.23 (p=.078) | -0.02 (p=.887) |
|  | B-ADL total score | -0.44 (p=.001) | -0.38 (p=.003) | 0.14 (p=.304) |
|  | NPI total score | 0.03 (p=.848) | 0.11 (p=.417) | -0.58 (p<.001) |
|  | DEMQOL proxy total score | 0.09 (p=.495) | -0.20 (p=.136) | 0.354 (p=.007) |

Key: BADL = Bristol activity of daily living scale; CDR = clinical dementia rating scale score; HADS = hospital anxiety and depression scale score; NPI = neuropsychiatric inventory score

*Appendix C: Scree plot from exploratory factor analysis of social functioning in dementia scale.*


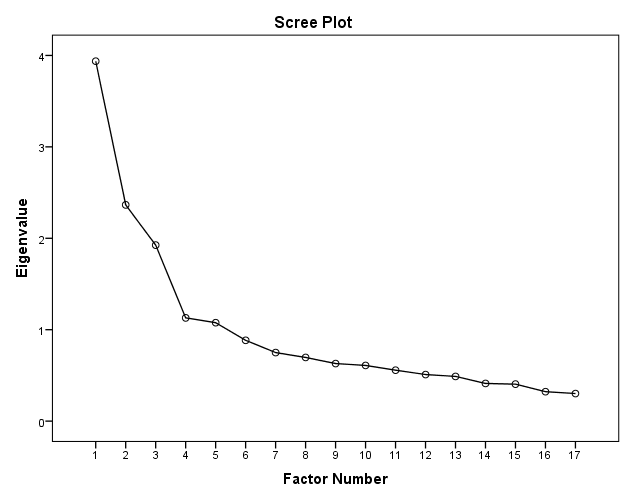

Supplement: Supplementary Appendices A–C [file mmc1.docx]
